# The Relationship between Parental Perception of Neighborhood Collective Efficacy and Physical Violence by Parents against Preschool Children: A Cross-Sectional Study in a County of China

**DOI:** 10.3390/ijerph16132306

**Published:** 2019-06-28

**Authors:** Haixue Wang, Jingqi Chen, Linjing Lyu

**Affiliations:** Institute of Child and Adolescent Health, School of Public Health, Peking University Health Science Center, Beijing 100191, China

**Keywords:** parental perception of neighborhood collective efficacy, physical violence, preschool children, risk factors, China

## Abstract

Children exposed to negative neighborhood environments are at high risk of experiencing violence. This study aimed to explore the effects of parental perception of neighborhood collective efficacy on parental physical violence (PV) to their preschool children in a county of China. A total of 1337 parents from nine kindergartens were recruited by the stratified random cluster sampling method. Data about parental PV behavior toward children during the past three months, parental perception of neighborhood collective efficacy, together with their attitudes towards the use of corporal punishment to discipline children, and demographic characteristics were collected. Their relationships were investigated by applying multivariable logistic regression models. Overall, 67.5% of the parents reported at least one form of PV during the past three months. The rates of minor PV (MPV) and severe PV (SPV) were 67% and 22.8%, respectively. The results of multivariate logistic regression showed that only social cohesion was associated with lower odds of parental PV and MPV behavior after controlling for covariates. The results suggest that neighborhood collective efficacy is associated with parental PV behavior against their children to some extent, but the effects differ according to the severity level of PV. Neighborhood social cohesion may have a positive role in reducing parental PV behavior in the county surveyed at present study.

## 1. Introduction

Physical violence (PV) against children is still a significant public health concern worldwide [1]. A meta-analysis showed that the life-time prevalence of any form of PV against child in China was about 36.6% [2], which was significantly higher than Stoltenborgh’s estimated international prevalence of 22.6% [1]. Children suffering from PV are at great risk for negative mental, physical, social and economic consequences throughout the course of their lives, bringing great burden to individuals, families, and societies [3,4,5,6].

Parental PV behavior against children is a complex social problem. Identifying significant risk factors is a crucial first step to formulate successful strategies for preventing parental physical violence against their children. Generally speaking, etiology of physical violence against children can be classified as developmental (the role of parent and child characteristics and processes), immediate interactional (parenting and parent-child interactional processes), and broader contextual factors (community, cultural, and evolutionary contexts) based on the developmental-ecological framework [7].

During the past years, many studies have focused their research on the former two aspects to understand this violent behavior [8,9]. As for the role of the latter in influencing the occurrence of physical violence, a growing number of studies had suggested that characteristics of neighborhoods are associated with parental PV behavior [10,11,12]. Among the studies that examined neighborhood characteristics and parental PV behavior, although studies found that negative community context, like community poverty, unemployment, and residential instability, may put parents at higher risks for child maltreatment [10,11,12], there are also some potentially modifiable neighborhood characteristics that may prevent child maltreatment, such as neighborhood collective efficacy [13,14,15].

Neighborhood collective efficacy is defined as a belief in social cohesion and as sharing expectations with neighborhood residents about intervention in the pursue of a common goal or to solve a community problem [16]. Its measurement combines social control (norms regarding appropriate behaviors and willingness to intervene) and social cohesion (mutual trust among neighbors) [16,17,18]. Collective efficacy has a variety of specific plausible mechanisms through which it could affect parental PV behavior against children. Studies found that neighborhoods with higher levels of collective efficacy are positively associated with residents’ perceptions of non-familial social support [19], and social support has been frequently proved to be linked with positive parenting behavior [20,21,22], which may contribute to the reduction in parents’ use of PV. However, parents who lived in neighborhoods with low levels of collective efficacy tend to experience more psychological distress [23,24], intimate partner violence [25], and substance abuse [26], all of which are risk factors for parental use of physical violence toward their children [27,28,29].

Currently, most studies involving one or two aspects of neighborhood collective efficacy have found that a lower level of collective efficacy was associated with higher rates of parental use of punitive child discipline [13,15,30,31,32,33,34]. However, not all results support this relationship [17,35]. Ma et al. [36] found that informal social control was not associated with lower odds of child protective services involvement, which was associated with parental spanking behavior. Kathryn et al. [14] demonstrated that neighborhood social cohesion was only associated with child neglect, but not any severity level of abuse. The mixed results suggested that further studies are needed to clarify our understanding of the neighborhood collective efficacy-parental PV behavior relationship.

Cross-cultural studies are of great significance to better understand a theory. The results above were all obtained by studies conducted in Western countries, and none of the evidence has been found to explore the relationship between neighborhood collective efficacy and parental PV behavior in China, a country with the rapid speed of economic reform and urbanization. While there is no empirical study on the relationship between neighborhood collective efficacy and parental PV behavior against children in China, based on the results of previous studies conducted in other countries, we hypothesize that, similar to Western countries, the neighborhood context in China may also play an important role in shaping parents’ PV behavior toward their children. In addition, China is a country where people are affected by the traditional culture “beating is caring and scolding is loving” and “spare the rod and spoil the child” [37]. Thus, under this special culture and development background, it is of great significance to explore the role neighborhood collective efficacy plays in shaping parental PV behavior against their children. The results may have great guide meaning and application value on interpreting and solving this complex social problem in China.

Early childhood years are of great significance to the well-being and success in adulthood [38,39]. However, evidence shows that children suffered the highest frequency of parental spanking during their preschool years [40]. A study conducted among preschool children in a county of Hunan Province in China found that, in the previous three months, there was a prevalence of physical violence against preschool children of 77.7% [41]. Considering the large negative effects of PV on children’s health development, it is of great significance to conduct a survey aiming at providing reference for a program of reducing parental PV behavior toward their preschool children.

Thus, in the present study, we aim to explore the possible inter-relationship between parental perception of neighborhood collective efficacy and parental PV behavior with a series of prospective logistic regression analyses. Our models examine the independent effects of neighborhood collective efficacy at preschool children’s parents on parental PV behavior; the simultaneous effects of collective efficacy and parents’ attitudes toward the use of corporal punishment to discipline children on parental PV behavior in a combined model, and the simultaneous effects of collective efficacy and parents’ attitudes toward the use of corporal punishment to discipline children in a full model that also controls for demographic characteristics which may have possible confounding associations with the predictors and the outcome. In addition, we also try to explore whether those associations differ according to the severity level of PV. We hypothesized that neighborhood collective efficacy would predict parental PV behavior in each model. We hope the results may contribute to gaining evidence to conduct prevention programs from a new perspective.

## 2. Methods

### 2.1. Study Sample

This was a cross-sectional study conducted in a county of Zhumadian City, Henan Province, a place located in the central area of China, during May 2018. This is an ordinary county with a population over 900,000. It includes three sub-districts located in the center of the county (urban areas), and 19 townships surround the central part of the county (rural areas). In total, 122 independent kindergartens have been set up in this county, with about 32,000 enrolled pre-school children. Among them, there are 44 kindergartens with about 12,000 enrolled pre-school children in urban areas, and 78 kindergartens with about 20,000 enrolled pre-school children in rural areas.

Stratified random cluster sampling method was used and parents from nine kindergartens, including four from the urban areas and five from rural areas were surveyed. At kindergartens with the number of children far beyond 300, three classes in each grade were selected, in total nine classes with about 300 children were selected. At kindergartens with the number of children around or less than 300, all the children were selected. For the selected children, one of their parents (father/mother) were invited to participate in the study with the task of completing a self-reported questionnaire.

The study was approved by the Peking University Institutional Review Board. Survey procedures were designed to protect respondents’ privacy by allowing for anonymous and voluntary participation, and they could withdraw from the survey at any time. An envelope containing the questionnaire was distributed to parents when they picked up their children after school, and the completed questionnaire was sealed in an envelope by each parent before returning it to the research team. Parents who filled in the questionnaire and returned them to the research team were considered to have agreed to participate in the investigation.

In total, 2121 questionnaires were sent to parents, and 1832 non-blank questionnaires were finally returned, with a participation rate of 86.4%. After excluding those questionnaires with missing data equal or surpassing one third, those that did not answer all of the eight items about parental PV, and those written by other parents, such as grandparents, aunts or sisters, a total of 1337 parents were involved in the study, with an effective rate of 73.0%.

### 2.2. Measures

#### 2.2.1. Key Outcome Variable: Physical Violence by Parents Against Children

The PV scale was developed based on items used in relevant publications [21,41,42,43,44,45], and has been used in surveys of preschool children’s parents [41,45] and hearing loss children’s parents [46]. The scale included 8 items as follows: (1) Pushed or shook a child; (2) pinched or scratched a child; (3) hit child’s buttocks with hand; (4) hit child’s hand, foot, back, arm or leg with hand; (5) hit child’s face or head with hand; (6) hit child’s buttocks with an object; (7) hit elsewhere (not buttocks) with an object; and (8) kicked a child with a foot or hit with a fist. For each item, parents were asked how often they acted the behaviors to their children during the past three months. The response categories included “never”, “1–2 times”,”3–5 times”, “6–10 times”, and “more than 10 times”. PV was defined as having one or more behaviors among the eight items during the past three months. At least one behavior happened among items (1)–(4) were defined as minor PV (MPV), and at least one behavior happened among the items (5)–(8) were defined as severe PV (SPV) [46,47]. The Cronbach’s α of the study in the elementary school pupils’ parents and hearing loss children’s parents were 0.85 [47] and 0.79 [46], respectively, and it was 0.86 in the present study.

#### 2.2.2. Key Independent Variables: Parental Perception of Neighborhood Collective Efficacy

Considering it is difficult to collect neighborhood level collective efficacy, this study only measured neighborhood collective efficacy relied on self-reported information from individual residents. Assessment of collective efficacy derived from previous study by Sampson et al. [16], and reflect a combination of factors related to both informal social control and social cohesion in a neighborhood.

The informal social control subscale includes four items and accesses parental perceptions of the likelihood that neighbors would intervene if children skipped school, sprayed graffiti, or disrespected an adult, if there were a fight. Responses were rated on a five-point Likert scale from very likely possible to very unlikely. Each person’s responses to these questions were averaged to provide an overall individual level informal social control (possible range 1–5), and two levels of informal social control were divided based on the score. The score of 0–3.9 was defined as middle/low level, 4–5 was defined as high level.

The social cohesion subscale includes four items and were used to measure parental perception of the extent to which their neighbors are helpful, close-knit, trustworthy, and share values. Responses were rated on a five-point Likert scale from strongly approve to strongly disapprove. Each person’s responses to these questions were averaged to provide an overall individual level social cohesion (possible range 1–5), and two levels of social cohesion were divided based on the score. The score of 0–3.9 was defined as middle/low level, 4–5 was defined as high level.

In total, the Cronbach’s α of the neighborhood collective efficacy scale in this study was 0.77, the Cronbach’s α of the informal social control and social cohesion subscales were both 0.82. More information about the reliability and validity of the scales, please see the Supplementary Materials (Tables S1 and S2).

#### 2.2.3. Covariates

##### Parents’ Attitudes Toward the Use of Corporal Punishment to Discipline Children

The scale including five items is a brief instrument designed to measure parents’ views agreed with the corporal punishment. Parents were asked whether they approve the view. Response choices included strongly disapprove, disapprove, unsure, approve, strongly approve. The correct attitudes of corporal punishment was defined as disapproving all the five views in this scale, otherwise, it was incorrect if the parent approved or not sure at least one items, the Cronbach’s α of the scale was 0.85 in primary school students’ parents [46,47], and it was 0.82 in this study.

The items of the scale are as follows: (1) In order to keep children follow the rules, it is necessary to hit; (2) in order to enable children to discriminate between good and bad, it is necessary to hit them; (3) spare the rod, spoil the child; (4) parents can hit their children when their child is talking back or crying; and (5) it is family affair that parents hit and scold their children, and outsiders should not intervene.

##### Demographic Characteristics

Parents were also asked to provide information about them, including gender, age, educational attainment, marital satisfaction, family economic situation in the local area, and childhood experiences of PV victimization before 16 years old. They were also asked to report the characteristics of their children, including child age, gender, and only-one child or not.

### 2.3. Data Analysis

Data were analyzed by SPSS software (version 20.0; SPSS Inc., Chicago, IL, USA). Descriptive statistics on the characteristics of the study population as well as the prevalence of PV, MPV, and SPV were reported, and Pearson chi-square test was performed to test the differences of the rate among different characteristics of the participants.

Multivariate logistic regression models were employed to examine the associations between parental perception of neighborhood collective efficacy and the occurrence of PV, MPV, and SPV. Every full model includes three explanatory variables—parental perception of neighborhood informal social control, parental perception of neighborhood social cohesion and parental attitudes toward the use of corporal punishment to discipline children—and demographic characteristics as covariates.

## 3. Results

The prevalence of parental self-reporting at least one form of PV during the past three months was 67.5%, and the prevalence of MPV and SPV was 67.0% and 22.8%, respectively. Descriptive data of respondents’ characteristics and bivariable associations with parental PV behavior against children are presented in Table 1. Among the 1337 parents, 80% of them were mothers, the mean age of them was 32.4 years (range, 22–66), and the mean age of their children was 4.2 years (range, 2–8). Bivariate analysis indicated significantly higher rates of any severity level of PV among rural parents than urban parents. Parents who were mothers, younger than 35 years, with lower educational level, unsatisfied with their marriage, and suffered physical violence before 16 years were also more likely to use any severity level of PV to discipline their children. Additionally, children in the low age group were at a higher risk of suffering PV or MPV from parents, and boys were more likely to be treated with SPV.

Descriptive statistics for variable of parental perception of neighborhood collective efficacy is provided in Table 2. The mean parental perception of informal social control and social cohesion value was 3.38 and 3.90, respectively. In bivariable analysis, both lower parental perception of informal social control and social cohesion were associated with a higher rate of parental PV or MPV behavior (*p* < 0.001) (Table 3), significant association between parental perception of neighborhood collective efficacy and parental SPV behavior was only observed in parental perception of social cohesion. In addition, we also found that more than half (52.6%) of the parents hold wrong attitudes toward the use of corporal punishment to discipline children, and those parents were at higher risks of conducting PV behavior to their children (*p* < 0.001) (Table 3).

The results of multivariate logistic regression models predicting parental PV behavior are shown in Table 4. The final column presents the full model inclusive of both parental perceived neighborhood collective efficacy and parents’ attitudes toward the use of corporal punishment to discipline children, and the covariates (Model 3). The results showed that one aspect of collective efficacy (i.e., social cohesion) and parents’ attitudes toward the use of corporal punishment to discipline children predicted parental PV and MPV behavior toward their children after controlling for other demographic characteristics showed in Table 1. Lower levels of social cohesion increased the odds of parental use of PV by 60% (95% CI 1.169–2.191) while parental attitudes toward the use of corporal punishment to discipline children was associated with a 236% increase in the odds of parental use of PV (95% CI 2.478–4.551), similar results were also observed in parental MPV behavior. However, none of the two aspects of parental perception of neighborhood collective efficacy were associated with parental SPV behavior, and parents who hold wrong attitudes toward the use of corporal punishment to discipline children were 2.85 times more likely to use SPV behavior to their children.

## 4. Discussion

The association between neighborhood collective efficacy and parental PV behavior against children has been widely studied in Western countries, but little is known about whether neighborhood environment matters for parents’ PV behavior against children in China. This study tried to fill this gap by conducting a cross-sectional survey among preschool children’s parents in China. It contributes to the literature by exploring the neighborhood effect in a developing country where tolerance of PV to discipline children still exists in some people.

The results of this study showed that a higher level of one aspect of neighborhood collective efficacy (i.e., social cohesion) is associated with lower odds of parental PV and MPV behavior against their children, even after controlling for parental attitudes toward the use of corporal punishment to discipline children. However, no significant associations were observed between parental perception of neighborhood collective efficacy and parental SPV behavior. These findings, to some extent, support existing literature on the role of neighborhoods collective efficacy in shaping parental PV behavior toward their children [13] and highlight the importance of conducting multilevel intervention strategy to prevent parental PV behavior toward their children, but the effects of neighborhood may differ according to the severity level of parental PV behavior.

Associations between neighborhoods with low social cohesion and parental PV behavior to their children accord with previous studies conducted in the U.S. [13,34,36]. The mechanism of this finding could be that higher level of social support acquired by living in neighborhoods with higher level of social cohesion can help reduce parents’ PV behavior toward their children. In addition, previous studies have also found that residents’ higher perceptions of mutual trust among neighbors increase one’s willingness to intervene in the situation of neighborhoods’ child abuse and neglect behavior [48,49]. Thus, the result suggests that promoting neighborhood relationships among residents that support the needs and challenges of parents with young children [50] can help ensure children’ safety.

However, when we analyzed parents’ PV behavior according to the severity level of PV, the results showed that significant effects of neighborhood social cohesion were only observed in parents’ MPV behaviors, but parents’ attitude toward corporal punishment can affect both parents’ MPV and SPV behavior toward their children. Considering the old traditional culture that “beating is caring and scolding is loving” in China, it is easy to understand why attitudes play an important role in shaping parents’ parenting behavior. However, the mechanism of why parental perception of neighborhood social cohesion could not affect parents’ SPV behavior toward their children still needs further study. One possible explanation that neighborhood social cohesion is not associated with parental SPV behavior is that according to our knowledge, parents’ SPV behavior usually happen when their children made serious mistakes, at this time, although many parents do not want to beat their children severely, they eventually perform this PV behavior due to the reason of lose control. In this situation, higher neighborhood collective efficacy could not play its role in reducing parents’ SPV behavior toward their children.

In addition, although the study found that the effect of parental perception of social cohesion was smaller than parental attitudes toward the use of corporal punishment to discipline children. These findings still provided support for the positive effects of social cohesion on parental PV behavior toward children, and supplemented the different effects of collective efficacy on PV among different countries. Thus, except conducting education programs aimed to improve parents’ awareness of PV and teach them new parenting skills to replace violence behavior, intervention programs should also pay attention to the importance of improving neighborhood social cohesion on preventing parental PV and MPV behaviors.

The results in this study that informal social control was not associated with parental PV behavior was contradictory to previous studies [13,34], but it was consistent with Yonas et al. [51] and Molnar et al. [35]. Emery and his colleague [15] suggested that the mixed results happen to some extent due to the reason that the measure of informal social control were about control of street crime and deviance, and social problems in public places, but not specific to child maltreatment. However, they thought that people willing to intervene against street crime does not mean they willing to intervene violence behind closed doors. Therefore, they suggested and developed a new measure of informal social control specific to child maltreatment, and their results found positive results of informal social control and parents’ PV behavior toward their children. Thus, further study should be conducted to examine the associations between informal social control and parental PV behaviors using the measure of informal social control specific to child maltreatment.

Considering the participants surveyed were from nine different schools in the county, clustering effect may exist among different schools, which might affect the relationship between parental PV behavior and neighborhood collective efficacy. Thus we conducted a null model by using multilevel logistic regression models to confirm whether this clustering effect existed. However, no significant results were observed, suggesting that the prevalence of PV differs far more between parents than across schools. Thus, we only reported the univariate and multivariate results. However, China is a country with a vast territory and the development levels varies according to regions, which could lead to significant differences of neighborhood processes in other areas. Therefore, the results may not be able to generalize to other areas of China. Further studies involving larger sample and different levels of cities are urgently needed to examine the relationships between neighborhood collective efficacy and parental PV behavior toward their children.

### Limitations

There are several limitations which should be noted here. Firstly, this study only measures parental perception of the neighborhood collective efficacy. However, parents who conducted PV behavior to their children may have more negative perceptions of their neighborhoods than their counterparts, if this is true for our study to some extent, the results of the relationship between neighborhood collective efficacy and parental PV behavior may be conservatively estimated. Thus, there is a need for research that explores the relationship between neighborhood-level collective efficacy and parental PV behavior adjusted for parental perception of collective efficacy.

Secondly, neighborhood structural characteristics (e.g., rates of poverty, unemployment) were not measured and controlled in this study, this may also influence the result of our study to some extent. On the one hand, negative neighborhood structural characteristics have been consistently proved to be risk factors of child maltreatment [13]. On the other hand, neighborhood structural characteristics may influence neighborhood social processes (e.g., collective efficacy) [12], and neighborhood collective efficacy partially mediates the association of neighborhood structural factors and people’s health [18]. That is, even after the influence of collective efficacy has been taken into consideration, neighborhood structural characteristics remains a direct predictor of many outcomes [52]. Thus, further studies should also take neighborhood structural characteristics into consideration when exploring the relationship between neighborhood characteristics and parental PV behavior. In addition, the measurement of family economic situation should also be improved.

Thirdly, the study population was very limited. Although we adopted stratified random cluster sampling method in the study area, it still mainly relied on parents within an ordinary county where urban and rural residents may share almost the same level of socioeconomic characteristics. However, considering the differences of urban and rural areas in China, to better understand the effect of neighborhood collective efficacy on parental PV behavior, it is in urgent need to conduct studies involving more cities with different developing levels.

Lastly, the rate of PV was possible to be underestimated in this study, on the one hand, the data relied on parents self-reported PV behaviors to their children, some parents may not report the actual events and it may cause self-report bias. On the other hand, only one parent in each family participated in this study, and parents who were selected to answer the questionnaire may have less PV behaviors than their partners.

Although these limitations exist, to our knowledge, this is the first study conducted in China to explore the associations between neighborhood collective efficacy and parental PV behavior to their children and we also explore this effect according to the severity level of parental PV behavior. This study considered both parental perception of neighborhood level risk factors, such as neighborhoods with low social cohesion, and parental level risk factors, such as wrong attitudes toward the use of corporal punishment to discipline children, which was affected by Chinese traditional culture. In addition, our analysis model provides separate, but simultaneous estimates of the associations of parental perception of neighborhood collective efficacy, and parental attitudes toward the use of corporal punishment to discipline children with parents’ PV behavior to their children. Further, although this study was only performed in a county of China, the respondents were recruited by using stratified random cluster sampling method, to some extent, the results could reflect the situation in other counties with the similar level.

## 5. Conclusions

Neighborhood collective efficacy is associated with parental PV behavior against children to some extent in the county surveyed at present study, but the effects may differ according to the severity level of PV. This study suggested that professionals who devote to child protection should focus on not only conducting intervention programs aimed to improve parents parenting skills and change their awareness about PV, but should also address strategies for promoting trusting and supportive relationships with neighbors to enhance social cohesion in their communities.

## Figures and Tables

**Table 1:** Respondents’ characteristics and bivariable associations with parental PV behavior to children.

Variables	n (%)	PV		MPV		SPV	
		n (%)	*χ*^2^ Value	n (%)	*χ*^2^ Value	n (%)	*χ*^2^ Value
Areas			6.369 *		6.341 *		19.663 ***
Urban	662 (49.5)	425 (64.2)		422 (63.7)		117 (17.7)	
Rural	675 (50.5)	477 (70.7)		474 (70.2)		188 (27.9)	
Gender			30.998 ***		30.462 ***		5.142 *
Father	267 (20.0)	142 (53.2)		141 (52.8)		47 (17.6)	
Mother	1070 (80.0)	760 (71.0)		755 (70.6)		258 (24.1)	
Parental age, years			20.645 ***		21.495 ***		10.363 **
>35	304 (24.8)	176 (57.9)		174 (57.2)		50 (16.4)	
≤35	924 (75.2)	664 (71.9)		661 (71.5)		235 (25.4)	
Missing	109						
Education level			7.195 **		7.207 **		16.843 ***
High school or higher	637 (48.3)	409 (64.2)		406 (63.7)		115 (18.1)	
Less than high school	682 (51.7)	485 (71.1)		482 (70.7)		188 (27.6)	
Missing	18						
Marital satisfaction			10.179 **		9.067 **		15.325 ***
Satisfied	1112 (84.1)	733 (65.9)		729 (65.6)		233 (21.0)	
Unatisfied	210 (15.9)	162 (77.1)		160 (76.2)		70 (33.3)	
Missing	15						
Family economic situation			0.119		0.080		0.014
Medium or rich	1168 (88.8)	786 (67.3)		781 (66.9)		265 (22.7)	
Poor	147 (11.2)	101 (68.7)		100 (68.0)		34 (23.1)	
Missing	22						
Parental PV experience before 16			137.339 ***		140.108 ***		26.486 ***
No	389 (29.3)	172 (44.2)		169 (43.4)		53 (13.6)	
Yes	938 (70.7)	725 (77.3)		722 (77.0)		250 (26.7)	
Missing	10						
Child gender			2.243		3.017		4.017 *
Girl	646 (48.3)	423 (65.5)		418 (64.7)		132 (20.4)	
Boy	691 (51.7)	479 (69.3)		478 (69.2)		173 (25.0)	
Child age, year			6.149 *		6.633 *		1.042
≥6	204 (15.9)	132 (64.7)		132 (64.7)		48 (23.5)	
4–5	685 (53.3)	447 (65.3)		442 (64.5)		164 (23.9)	
≤3	395 (30.8)	285 (72.2)		284 (71.9)		84 (21.3)	
Missing	53						
Only-one child family			0.256		0.303		1.656
Yes	161 (12.3)	106 (65.8)		105 (65.2)		30 (18.6)	
No	1144 (87.7)	776 (67.8)		771 (67.4)		265 (23.2)	
Missing	32						

Note: * *p* < 0.05, ** *p* < 0.01, *** *p* < 0.001. PV: physical violence; MPV: minor physical violence; SPV: severe physical violence.

**Table 2:** Descriptive statistics for variable of parental perception of neighborhood collective efficacy.

Variables	Mean	S.D.	Min	Max
Parental perception of informal social control	3.38	0.88	1.00	5.00
High-level	4.31	0.35	4.00	5.00
Middle/Low-level	2.95	0.70	1.00	3.75
Parental perception of social cohesion	3.90	0.61	1.00	5.00
High-level	4.44	0.40	4.00	5.00
Middle/Low-level	3.48	0.37	1.00	3.75

Note: S.D. stands for standard deviation.

**Table 3:** Descriptive statistics for variable of parental perception of neighborhood collective efficacy.

Variables	n (%)	PV		MPV		SPV	
		n (%)	*χ*^2^ value	n (%)	*χ*^2^ value	n (%)	*χ*^2^ value
Parental perception of informal social control			21.790 ***		22.940 ***		0.096
High-level	416 (31.7)	245 (58.9)		242 (58.2)		93 (22.4)	
Middle/Low-level	895 (68.3)	643 (71.8)		640 (71.5)		207 (23.1)	
Parental perception of social cohesion			40.194 ***		38.930 ***		5.330 *
High-level	567 (44.0)	332 (58.6)		330 (58.2)		113 (19.9)	
Middle/Low-level	721 (56.0)	542 (75.2)		538 (74.6)		183 (25.4)	
Parents’ attitudes toward the use of corporal punishment to discipline children			116.843 ***		113.901 ***		63.034 ***
Right	631 (47.4)	334 (52.9)		332 (52.6)		83 (13.2)	
Wrong	700 (52.6)	565 (80.7)		561 (80.1)		220 (31.4)	

Note: ** *p* < 0.01, *** *p* < 0.001. PV: physical violence; MPV: minor physical violence; SPV: severe physical violence.

**Table 4:** Multivariate logistic regression models with different severity level of PV as outcomes.

Physical Violence	Variables	Model 1 ^a^ OR (95% CI)	Model 2 ^b^ OR (95% CI)	Model 3 ^c^ OR (95% CI)
PV	Parental perception of informal social control	1.47 (1.133–1.903) **	1.41 (1.075–1.852) *	1.25 (0.893–1.736)
	Parental perception of social cohesion	1.95 (1.517–2.496) ***	1.72 (1.326–2.234) ***	1.60 (1.169–2.191) **
	Parents’ attitudes toward the use of corporal punishment to discipline children		3.50 (2.712–4.509) ***	3.36 (2.478–4.551) ***
MPV	Parental perception of informal social control	1.50 (1.156–1.938) **	1.44 (1.100–1.891) **	1.27 (0.909–1.765)
	Parental perception of social cohesion	1.91 (1.488–2.443) ***	1.69 (1.301–2.186) ***	1.59 (1.159–2.171) **
	Parents’ attitudes toward the use of corporal punishment to discipline children		3.41 (2.652–4.394) ***	3.29 (2.432–4.455) ***
SPV	Parental perception of informal social control	0.96 (0.717–1.292)	0.90 (0.666–1.216)	0.86 (0.610–1.198)
	Parental perception of social cohesion	1.39 (1.055–1.843) *	1.23 (0.920–1.631)	1.00 (0.724–1.371)
	Parents’ attitudes toward the use of corporal punishment to discipline children		2.99 (2.238–3.994) ***	2.85 (2.069–3.928) ***

Note: * *p* < 0.05, ** *p* < 0.01, *** *p* < 0.001. ^a^ The multivariate analysis model examined the independent effects of parental perception of neighborhood collective efficacy. ^b^ The multivariate analysis model adjusted for parents’ attitudes toward the use of corporal punishment to discipline children. ^c^ The multivariate analysis model adjusted for parents’ attitudes toward the use of corporal punishment to discipline children and demographic characteristics showed in Table 1. PV: physical violence; MPV: minor physical violence. SPV: severe physical violence; CI: confidence interval; OR: odds ratio.

## Supplementary Materials

The following are available online at https://www.mdpi.com/1660-4601/16/13/2306/s1, Table S1: Results of the reliability analysis; Table S2: Results of the validity analysis.
