# Erratum: Wang, H., et al. The Relationship between Parental Perception of Neighborhood Collective Efficacy and Physical Violence by Parents against Preschool Children: A Cross-Sectional Study in a County of China. *Int. J. Environ. Res. Public Health* 2019, *16*, 2306

**DOI:** 10.3390/ijerph17010145

**Published:** 2019-12-24

**Authors:** Haixue Wang, Jingqi Chen, Linjing Lyu

**Affiliations:** Institute of Child and Adolescent Health, School of Public Health, Peking University Health Science Center, Beijing 100191, China; gwwanghaixue@126.com (H.W.); LLJ1160550662@163.com (L.L.)

The authors would like to correct the Cronbach’s α for the measures in the “Methods” section of their previous paper [1] as follows:

## Incorrect Cronbach’s α

1. 2.2.1. Key Outcome Variable: Physical Violence by Parents Against Children
  The last sentence: “…...respectively, and it was 0.86 in the present study.”
2. 2.2.3. Covariates
  Parents’ Attitudes Toward the Use of Corporal Punishment to Discipline Children
  The first paragraph’s last sentence: “…..., and it was 0.82 in the present study.”

## Correct Cronbach’s α

1. 2.2.1. Key Outcome Variable: Physical Violence by Parents Against Children
  The last sentence: “…...respectively, and it was 0.85 in the present study.”
2. 2.2.3. Covariates
  Parents’ Attitudes Toward the Use of Corporal Punishment to Discipline Children
  The first paragraph’s last sentence: “…..., and it was 0.81 in the present study.”

## References

[1] Wang H., Chen J., Lyu L. (2019). The Relationship between Parental Perception of Neighborhood Collective Efficacy and Physical Violence by Parents against Preschool Children: A Cross-Sectional Study in a County of China. Int. J. Environ. Res. Public Health.

