# Supplementary material for: The Relationship between Parental Perception of Neighborhood Collective Efficacy and Physical Violence by Parents against Preschool Children: A Cross-Sectional Study in a County of China
Source: Int J Environ Res Public Health. 2019 Jun 28;16(13):2306. doi: 10.3390/ijerph16132306 (PMC6651238; doi:10.3390/ijerph16132306)
Supplement: Supplementary file 1 [file ijerph-16-02306-s001.pdf]

Supplementary Materials

Results of the Reliability and Validity Analysis of the Neighborhood Collective Efficacy Scales

SPSS software (version 20.0; SPSS Inc., Chicago, IL, USA) was used to evaluate the reliability of the neighborhood collective efficacy scales, indictors including Cronbach's α (>0.80), corrected item-total correlation (CITC > 0.40) and Cronbach's alpha if item deleted (ID α < Cronbach's α). The analytic technique of confirmatory factor analysis (CFA) was used to assess the validity of the constructs of the neighborhood collective efficacy scales. Maximum likelihood estimation was used to fit the model. Standardized factor loadings (≥0.50), average variance extracted (AVE ≥ 0.50), and composite reliability (CR ≥ 0.70) were calculated to evaluated the construct validity of each scale. Indicators including incremental fit index (IFI > 0.90), goodness-of-fit index (GFI > 0.90), adjusted GFI (AGFI > 0.90), comparative fit index (CFI > 0.90), and the root-mean-square error of approximation (RMSEA < 0.08) were used to assess the fit of the model.

In total, there are nine items in the neighborhood collective efficacy scale, four items about informal social control, and five about social cohesion. However, we found that the Cronbach's α and other indicators about this scale will increase after deleting the reverse coded item about neighborhood get along in the social cohesion subscale when we calculated the reliability and validity of the scales. Thus, four items left in the social cohesion subscale. Here we reported the results of reliability and validity about the final scales.

The results of the reliability analysis were shown in Table S1. The Cronbach's α of the neighborhood collective efficacy scale was 0.77, and the Cronbach's α of the two subscales (neighborhood informal social control and neighborhood social cohesion) were both 0.82. From Table S1 we can see that, except of the CITC value of item of SC4 was a bit lower than 0.40, the other items' indicator values were all meet their standards, which means that the reliability of constructs of neighborhood collective efficacy were acceptable.

Table S1. Results of the reliability analysis.

Constructs and Items	Cronbach's α	CITC value	IDα
Neighborhood informal social control	0.82		
ISC1		0.53	0.73
ISC2		0.45	0.75
ISC3		0.59	0.72
ISC4		0.55	0.73
Neighborhood social cohesion	0.82		
SC1		0.50	0.74
SC2		0.47	0.75
SC3		0.46	0.75
SC4		0.24	0.78

The results of the validity analysis (CFA) were shown in Table S2. The AVE of the neighborhood collective efficacy of 0.59, the CR of the neighborhood collective efficacy scale was 0.92. From this table we can see that except for the item loading of SC3 being a bit lower than 0.50, other values of item loading, AVE, and CR were all greater than 0.50 and 0.70 for all constructs. In addition, the values of various fit indices indicated that the measurement models had a satisfactory fit.

Table S2. Results of the validity analysis.

Constructs and Items	AVE	CR	Item loading	*P*
Neighborhood informal social control	0.55	0.83		
ISC1			0.80	fixed
ISC2			0.58	<0.001
ISC3			0.80	<0.001
ISC4			0.78	<0.001
Neighborhood social cohesion	0.53	0.76		
SC1			0.91	fixed
SC2			0.93	<0.001
SC3			0.79	<0.001
SC4			0.46	<0.001

Fit indices of the measurement models: Relative chi-square = 178.702, P < 0.001; IFI (>0.90) = 0.966; GFI (>0.90) = 0.968; AGFI (>0.90) = 0.942; CFI (>0.90) = 0.966; RMSEA (<0.08) = 0.077 (standardized estimates).
